# Case-control study of the characteristics and risk factors of hot clot artefacts on 18F-FDG PET/CT

**DOI:** 10.1186/s40644-024-00760-1

**Published:** 2024-08-27

**Authors:** Jacques Dzuko Kamga, Romain Floch, Kevin Kerleguer, David Bourhis, Romain Le Pennec, Simon Hennebicq, Pierre-Yves Salaün, Ronan Abgral

**Affiliations:** 1 Nuclear Medicine Department, CHRU Brest, Boulevard Tanguy Prigent, Brest, France; 2 UMR Inserm GETBO 1304, University of Western Brittany, Brest, France

**Keywords:** Hot clot artifact, False-positive, FDG-PET pitfall

## Abstract

**Introduction:**

The pulmonary Hot Clot artifact (HCa) on 18F-FDG PET/CT is a poorly understood phenomenon, corresponding to the presence of a focal tracer uptake without anatomical lesion on combined CTscan. The hypothesis proposed in the literature is of microembolic origin. Our objectives were to determine the incidence of HCa, to analyze its characteristics and to identify associated factors.

**Methods:**

All 18F-FDG PET/CT retrieved reports containing the keywords (artifact/vascular adhesion/no morphological abnormality) during the period June 2021–2023 at Brest University Hospital were reviewed for HCa. Each case was associated with 2 control patients (same daily work-list). The anatomical and metabolic characteristics of HCa were analyzed. Factors related to FDG preparation/administration, patient and vascular history were investigated. Case-control differences between variables were tested using Chi-2 test and OR (qualitative) or Student’s t-test (quantitative).

**Results:**

Of the 22,671 18F-FDG PET/CT performed over 2 years, 211 patients (0.94%) showed HCa. The focus was single in 97.6%, peripheral in 75.3%, and located independently in the right or left lung (51.1% vs. 48.9%). Mean ± SD values for SUVmax, SUVmean, MTV and TLG were 11.3 ± 16.5, 5.1 ± 5.0, 0.3 ± 0.3 ml and 1.5 ± 2.1 g respectively. The presence of vascular adhesion (*p* < 0.001), patient age (*p* = 0.002) and proximal venous access (*p* = 0.001) were statistically associated with the presence of HCa.

**Conclusion:**

HCa is a real but rare phenomenon (incidence around 1%), mostly unique, intense, small in volume (< 1 ml), and associated with the presence of vascular FDG uptake, confirming the hypothesis of a microembolic origin due to probable vein wall trauma at the injection site.

## Introduction

18F-fluorodesoxyglucose positron emission tomography / computed tomography (18F-FDG PET/CT) is a functional imaging technique based on the study of glucose metabolism in cells. Although it is a whole-body scan, the analysis of the lungs remains fundamental in many contexts, not only in oncology. Indeed, FDG-PET/CT is now routinely recommended for the characterization of solid pulmonary nodules ≥ 8 mm and for the initial staging of non-small-cell lung cancer [1]. More recently, it can also be suggested for the management of infectious or inflammatory pathology, such as unknown chronic fever or sarcoidosis [2].

Numerous specific technical artifacts and potential pitfalls in the interpretation of PET/CT in the thoracic region, including normal variations in physiological uptake of 18F-FDG and benign conditions, have been well described [3]. Awareness of these pitfalls is crucial as they may lead to misinterpretation with consequences for patient management and therapeutic implications [4]. One cause of these false positives results, called “hot clot artefact” (HCa), is still poorly understood. HCa fulfils 3 criteria: (i) the presence of one or more focal pulmonary 18F-FDG uptake(s) without anatomical lesion on CT scan; (ii) the high level of visual and semi-quantitative metabolic activity of the foci; (iii) the disappearance or migration of foci on late or subsequent acquisition [4–6].

There is very little literature available on this subject, based mainly on the publication of several case reports, totaling approximately twenty cases (21 patients). Nevertheless, certain hypotheses have been proposed to explain this relatively rare phenomenon. Thus, pulmonary microvascular embolism due to clots formed at the 18F-FDG injection site as a result of the vascular lesion and the agglutinating nature of FDG is the most plausible mechanism, as some authors have reported para-venous injection, rapid injection or blood aspiration into the injector system [4, 7–11].

Such as a background, our aims were to determine the incidence of hot clot artefact in a large case-control PET/CT study, to analyze its 18FDG uptake characteristics and to identify its potential associated factors.

## Materials and methods

### Design

This is a single-center retrospective observational case control study conducted in the Nuclear Medicine Department of Brest University Hospital between June 2021 and June 2023. The study was conducted in accordance with the Declaration of Helsinki and was approved by the French Advisory Committee on Information Processing in Health Research (CCTIRS).

### Population

All patients who underwent a 18F-FDG PET/CT during the 2-year inclusion period were analysed, regardless of indication. First, examination reports available in the radiology information system (Xplore, EDL, Paris, France) were queried using an AI word recognition algorithm with the terms “artefact” and/or “vascular adhesion” and/or “no morphological abnormality”. All selected files were reviewed to authenticate HCa cases, defined as the presence of one or more focal pulmonary 18F-FDG uptake(s) without anatomical lesion on CT scan and disappearance of the focus or no appearance of pathological lesion on a subsequent scan. Finally, 2 control patients per case were included as those managed immediately before or after the selected case on the daily work list and using the same examination modality.

### 18F-FDG PET/CT procedure

All 18F-FDG PET/CT images were acquired on two digital Biograph Vision 600 PET/CT scanner systems (Siemens Healthineers, Knoxville, TN, USA) with the same technical settings.

Standard patient preparation included at least 4 h fasting and serum blood glucose level < 7 mmol/L prior to intravenous injection of approximately 3 MBq/kg (0.08 mCi/kg) of FDG by a nuclear medicine technologist (NMT) via a catheter or a permanent device (implantable chamber, PICC line or midline). After injection, patients remained in a quiet room for approximately 60 min before acquisition.

At first, CT scan was obtained just after injection of intravenous iodine contrast agent (1.5 mL/kg), unless contraindicated. The CT consisted in a 64-slice multidetector-row spiral scanner with the following parameters : 110 kVp tube voltage (automatic modulation carekV^®^); 80 refmAs effective tube current with automatic dose modulation (care4D^®^); 0.5 s rotation time; 19.2 mm total collimation width ; pitch 1, 512 matrix size, 0.98 × 0.98 mm pixels; 2 mm slices thickness.

Then, PET data were acquired in in the craniocaudal direction using a whole-body protocol (2 min per step) and were reconstructed using an iterative ordered subset expectation maximization (OSEM) algorithm (True X^®^ = point spread function (PSF) + time of flight (TOF) acquisition capabilities, 4 iterations, 5 subsets). Images were corrected for random coincidences, scatter and attenuation using the CT scan data and were smoothed with a Gaussian filter (full-width at half-maximum = 2 mm). The axial field of view was 263 mm and the overlap fraction was 49%. The reconstruction matrix was 440 × 440 voxels and the voxel size was 1.65 × 1.65 × 1.65 mm.

### Image analysis

Hot clot artifacts (HCa) were visually characterized in terms of number (single or multiple) and location (right or left; lower lobe (LL) or middle lobe (ML) or upper lobe (UL), peripheral or intermediate or proximal).

Tracer uptake was determined using SUVs, calculated according to the following formula: SUV = tissue radioactivity concentration [kBq/mL] / [injected dose (kBq) / patient weight (g)]. Various PET parameters were analyzed for each HCa using MIM software (MIM Software Inc., Cleveland, United States): SUVmax and SUVmean, corresponding to the maximum and average values of SUV respectively; MTV (metabolic target volume), defined as the summed volume in millilitres (mL) measured using an image gradient-based method (PET EDGE™) [12]; TLB (total lesion burden) in grams (g), defined as MTV x SUVmean.

### Data collection

A different set of data was collected for each case and control patient, including: (i) clinical characteristics [gender (M/F), age, weight, height, blood glucose level, active cancer defined as patient with a history of known cancer who had not achieved a complete response for at least 6 months at the time of the PET-CT (yes/no), anticoagulant treatment or antiplatelet drug (yes/no), and previous history of venous thrombosis or pulmonary embolism (yes/no)]; (ii) FDG administration [venous access (proximal/distal), permanent device (yes/no), NMT in charge, injected activity, time between 18F-FDG injection and image acquisition, iodinated contrast administration (yes/no)]; (iii) imaging procedure [PET machine (PET1/PET2), FDG vessel adhesion at injection site defined as venous linear uptake (yes/no), FDG extravasation into soft tissues (yes/no)].

### Statistics

Statistical analyses were performed using EpiInfo software version 7.2.6.0.

Descriptive statistics were used to characterize the cohort. Qualitative variables were presented as number (n) and percentage (%). The association between dichotomous categorical variables and the presence of the hot clot artifact was measured by the odd ratio (OR) with a 95% confidence interval (95%CI). Significant differences were assessed using chi-2 or Fisher exact test. Quantitative variables were expressed as mean ± standard deviation (SD) and compared in both case and control groups using Student t test. The level of significance was *p* < 0.05.

## Results

### Population

Among the 22,671 18F-FDG PET/CT scans performed in our department between June 2021 and June 2023, 211 patients (98 M/113F, mean age ± SD 62.2 ± 15.4 years) had at least one pulmonary hot clot artefact, corresponding to an incidence of 0.94%. For further analysis of potential associated factors, 422 controls were selected, i.e. 2 per case.

**Fig. 1:**
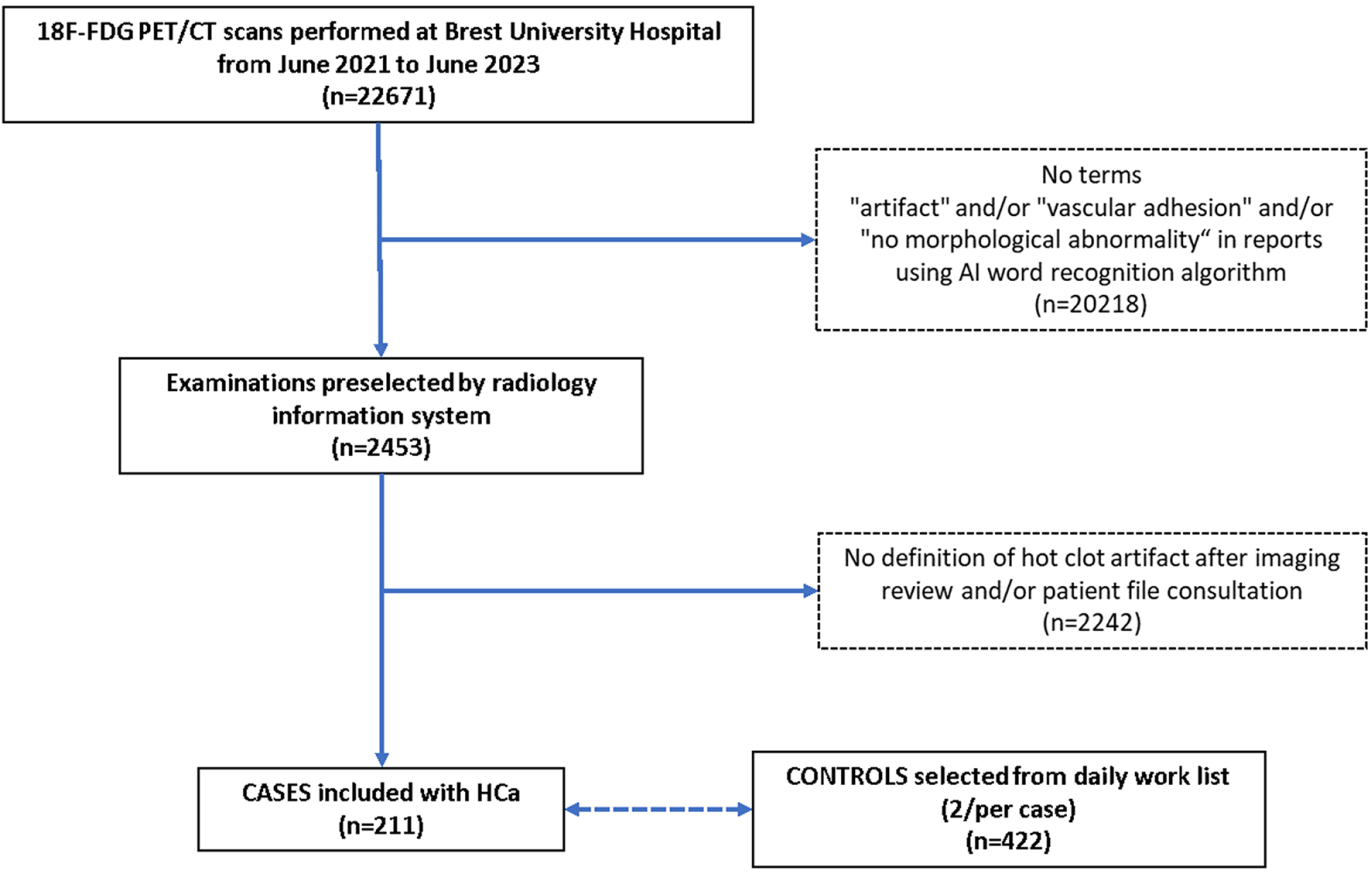
Flowchart of case-control patients’ selection

The selection of case-control patients is described in the flowchart (Fig. 1).

### Hot clot artifact description

**Fig. 2:**
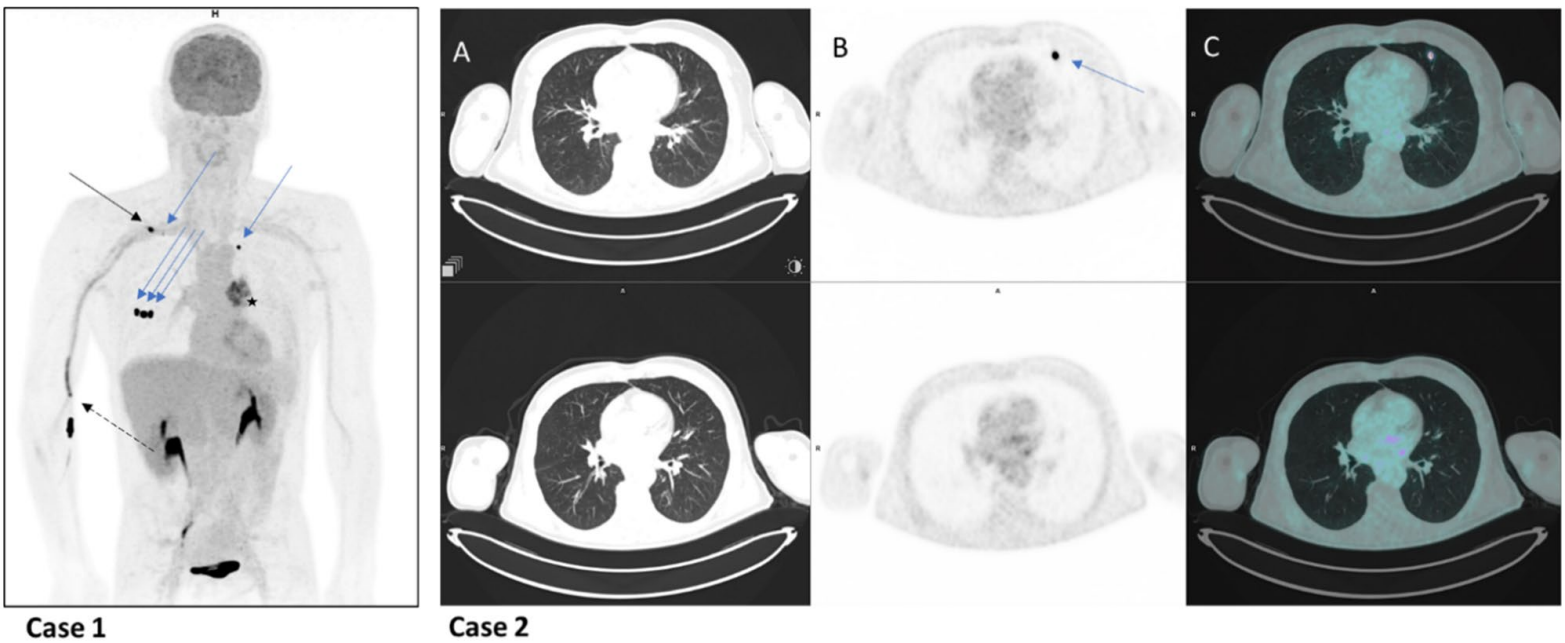
Presentation of 2 illustrative cases of HCa

HCa were single, double or quintuple in 206 (97.6%), 4 (1.9%) and 1 case (0.5%) respectively, and were located in the right lung 112 times (51.1%) (58 in UL, 19 in ML and 35 in LL) and in the left lung 107 times (48.9%) (68 in UL and 39 in LL). The focus was peripheral (less than 2 cm from the pleura or fissure), proximal (less than 2 cm from the hilum) or intermediate (others) in 165 (75.3%), 23 (10.5%) and 31 (14.2%) cases respectively (Fig. 2).

#### Case 1:

a 54-year-old patient underwent 18F-FDG PET scan as part of the staging of a left lung neoplasm. The MIP image showed FDG avidity of the tumour (star), FDG vascular uptake in the elbow and right arm (dotted black arrow), lymph node uptake in the right subclavicular region (black arrow), and 5 lung foci (blue arrows), 3 peripheral sub-scissural foci in the middle lobe, 1 peripheral sub-pleural foci in the left upper lobe, and 1 peripheral sub-pleural foci in the right upper lobe) without anatomical lesions opposite, corresponding to a quintuple case of Hca.

#### Case 2:

a 60-year-old patient with oral squamous cell carcinoma underwent 18F-FDG PET scans for staging (top row) and follow-up (bottom row). Focal FDG uptake in the peripheral subpleural region of the left upper lobe (blue arrow) on PET (B) and fused PET-CT images (C) with no CT abnormalities (A) disappeared on the second scan, confirming a case of HCa.

The mean values ± SD [Range] of SUVmax, SUVmean, MTV and TLG were 11.3 ± 16.5 [0.9–142.0], 5.1 ± 5.0 [0.7–35.6], 0.3 ± 0.3 ml [0.1–1.5] and 1.5 ± 2.1 g [0.2–18.8], respectively. Only 3/217 MTV values (1.4%) were greater than 1 ml.

### Associated factors

#### Clinical characteristics

There was no significant difference in clinical characteristics between case and control patients (Table 1), except for age (mean ± SD 62.2 ± 15.4 vs. 65.9 ± 13.8, *p* = 0.002).

**Table 1:** Distribution of case controls by clinical characteristics

Clinical characteristics	Cases (*n* = 211)	Controls (*n* = 422)	OR [95%CI]	*p*
**Sex**				
Male	98 (46.5)	194 (46.0)	1.02 (0.73–1.43)	0.910
Female	113 (53.6)	228 (54.0)		
**Age** (year)	62.2 ± 15.4	65.9 ± 13.8		0.002
**Weight** (Kg)	69.8 ± 15.9	71.0 ± 16.8		0.374
**Blood glucose level** (mmol/L)	5.7 ± 1.0	5.8 ± 1.2		0.124
**Active cancer**				
Yes	132 (62.6)	275 (65.2)	0.89 (0.63–1.27)	0.519
No	79 (37.4)	147 (34.8)		
**Anticoagulant treatment**				
Yes	29 (13.7)	51 (12.1)	1.16 (0.70–1.89)	0.773
No	143 (67.8)	297 (70.4)		
NA	39 (18.5)	74 (17.5)		
**Antiplatelet drug**				
Yes	34 (16.1)	68 (16.1)	1.00 (0.63–1.56)	0.997
No	139 (65.9)	279 (66.1)		
NA	38 (18.0)	75 (17.8)		
**Previous history of VTE**				
Yes	11 (5.2)	27 (6.4)	0.81 (0.38–1.64)	0.818
No	162 (76.8)	323 (76.5)		
NA	38 (18.0)	72 (17.1)		

NA = not available, VTE = venous thromboembolism

### FDG administration

Venous access (proximal vs. distal vs. permanent device) was associated with the occurence of HCa (*p* = 0.001). The distribution of case controls by FDG administration is shown in Table 2.

**Table 2:** Distribution of case controls by FDG and iodinated contrast administration

18F-FDG administration	Cases (*n* = 211)	Controls (*n* = 422)	OR [95%CI]	*p*
**Venous access**, n= (%)				
Proximal^1^	199 (94.3)	356 (84.4)	3.13 (1.67–7.14)	< 0.001
Distal^2^	9 (4.3)	57 (13.5)	0.29 (0.13–0.59)	< 0.001
Permanent device	3 (1.4)	9 (2.1)		
**NMT in charge** (*n* = 47)				0.994
**Injection-acquisition delay** (min)	60.6 ± 3.2	61.0 ± 4.6		0.265
**Injected activity** in MBq (mCi)	206.8 ± 47.8 (5.6 ± 1.3)	211.4 ± 49.1 (5.7 ± 1.3)		0.266
**Iodinated contrast administration**				
Yes	145 (68.7)	268 (63.5)	1.26 (0.89–1.80)	0.1941
No	66 (31.3)	154 (36.5)	0.79 (0.56–1.13)	

NMT = nuclear medicine technologist

^1^elbow, arm; ^2^forearm, wrist, hand, foot

### Imaging analysis

There was no difference in FDG extravasation into soft tissues between case controls, in contrast to FDG venous linear uptake at injection site on images, which was more frequent in the HCa case group than in the control group (64.9% vs. 42.2%, respectively; OR = 2.56 95%CI 1.79–3.70, *p* < 0.001) (Table 3).

**Table 3:** Distribution of case controls by imaging procedure

Image data	Cases (*n* = 211)	Controls (*n* = 422)	OR [95%CI]	*p*
**Vascular adhesion or FDG extravasation**	137 (64.9)	178 (42.2)	2.56 (1.79–3.70)	< 0.001
**PET machine**				0,736
PET 1	105 (49.8)	204 (48.3)		
PET 2	106 (50.2)	218 (51.7)		

## Discussion

Our results showed an incidence of pulmonary hot clot artifact (HCa) on 18F-FDG PET/CT of 0.94% (211/22671 scans for a total of 219 HCa) confirms the idea of a rare phenomenon. However, it has to be considered as a pitfall for physicians when interpreting images. Only Hany et al. found comparable results (*p* = 0.2 with X^2^ statistical test), reporting an artifact in 3 patients out of 750 examinations carried out over a 9-months period, i.e. a frequency of 0.4% [7]. To the best of our knowledge, this is the largest series investigating the incidence of HCa, as the literature on this subject is sparse and mostly consists of case reports [4, 5, 7–11, 13] (Table 4).

In our series, the HCa was almost exclusively single (206/211 = 97.6%). This finding is in accordance with the literature, as 19 of 21 (90.4%) published cases reported a single artifact. At most, we have showed an atypical example of a quintuple artifact in the same patient, as described by Ha et al. We found a balanced distribution of artifacts between the 2 lungs (51.1% versus 48.9%), redressing with a large population sample the predominance in the right lung (65%) extracted from the literature (12 patients, 17 artifacts). In our results, HCa were subpleural in approximately ¾ of the cases (75.3%), showing a tropism of the artifact for the peripheral region of the lung where the blood vessels are of smaller caliber and supporting the theory of a microscopic phenomenon an embolic origin of the artifact [4, 7–9, 11].

Regarding the metabolic characteristic of HCa, we found a high mean SUVmax 11.3 but with a large range [0.91 to 145], as calculated from data of 12 cases of literature (mean SUVmax ± SD = 40.6 ± 49.1 with a maximum of 185.1 and a minimum of 3.4) [4, 8, 10, 11, 13]. These findings demonstrate very high SUVmax values especially for possible lesion sizes below the spatial resolution of CT, as already suggested in several case report [4, 9, 13]. However, this very high variability of SUV parameters does not allow in current practice the use of a threshold to distinguish an artifact from a pathological lesion prior to its morphological expression. Nevertheless, its volume could be an interesting tool. Indeed, the mean MTV ± SD was 0.3 ± 0.3 ml in our series; and interestingly, 99% of them (216/219) presented a MTV lower than 1 ml. This again confirms that this artifact is a very low-volume phenomenon, such as micro-embolism. Therefore, a MTV value < 1 ml could be added as a new criterion for defining hot clot artifacts, avoiding repeat examinations (18F-FDG PET/CT or chest CT), thus limiting health care costs and improving patient management (consequences of misinterpretation, radiation exposure).

In our results, we found a significant statistical association between the presence of FDG vascular adhesion at the injection site (64.9% of cases vs. 42.2% of controls) and the presence of a hot clot artifact (OR = 2.56, 95%CI 1.79–3.70; *p* < 0.0001). This correlation favors an embolic origin, as we imagine that the stasis of the radiopharmaceutical at the injection site probably reflects trauma to the vein wall, making it likely that a hot clot formed and migrated towards the lung. This hypothesis already been raised in the literature. In fact, Sánchez-Sánchez et al. observed the presence of 18F-FDG extravasation in 3 of their 4 reported patients [11]. In addition, Farsad et al. described a para-venous injection in the 4 cases they reported [10]. The migration or disappearance of the HCa on late or subsequent scans and the absence of clinical consequences for all the 21 cases published are consistent with this micro-embolic origin [4, 5, 7–11, 13]. Moreover, regarding patient preparation, the venous proximal access was significantly higher in cases than in controls (94.3% of versus 84.4% of controls, *p* = 0.0012). This result may seem paradoxical, as distal veins are thinner and more fragile, and therefore probably at risk of HCa. One explanation might be that the systematic use of small-caliber catheters for distal access in our routine would ultimately be less traumatic and protect against this risk. Retrospectively, we verified the association HCa/FDG vessel adhesion on PET was independent of this venous access type. In addition, there was no association between the nuclear medicine technologist (NMT) responsible for patient management and the presence of the hot clot artifact (*p* = 0.994). This does not suggest an isolated problem of competence in venipuncture procedure, which appears to be fairly homogeneous within our department. Injection-acquisition time interval and injected activity were not correlated with the presence of hot clot artifact. However, these two parameters varied very little (about 60 min for the delay and 3 MBq (0.08 mCi)/kg body weight for the injected activity), as we routinely used procedural guidelines for PET imaging [14]. We found no statistical association between the PET machine used for acquisition (*p* = 0.736) and the presence of a HCa, but the 2 systems were of the same model with the same technical settings. However, a machine effect remains unlikely as the cases reported in the literature were published over a wide time interval (2003 to 2020). Therefore, differences related to technological advances in PET imaging (PSF + TOF acquisition capabilities, digital technology, etc…) during this period cannot be involved [15–19]. Finally, there was no statistical association between the administration of iodinated contrast and the presence of the warm clot artifact (*p* = 0.1941), even though both agents were injected into the same venous access, making a pro-coagulant interaction between FDG and iodinated contrast agent unlikely.

We choose a 1:2 case-control design using the daily PET work list to rule out an obvious lack of correlation between HCa occurrence and radiopharmaceutical production (chemical purity, batch number, etc…) or time dependence (seasonal period, pm vs. am, etc…). Our results showed that controls were on average older than cases (65.9 versus 62.2 years; *p* = 0.0021). At first sight, this may seem surprising, given that older people have a more fragile blood vessel system. On the contrary, one explanation could be that platelet function is better in younger people [20, 21]. The mean age of cases reported in the literature was 55.3 years (17 patients) [4, 5, 7–9, 11, 13]. In addition, other clinical characteristics were comparable between the 2 groups notably in terms of gender (*p* = 0.910), as reported in the literature (21 patients, 52% female and 48% male) [4, 5, 7–11, 13]. Finally, the presence of active cancer (*p* = 0.519), a history of deep vein thrombosis or pulmonary embolism (*p* = 0.818), anti-platelet drugs (*p* = 0.997) or anticoagulant treatment (*p* = 0.773) were not statistically associated with the presence of hot clot artifact. These factors were examined to identify potential circumstances associated with VTE that may or may not put patients at risk of thrombus formation.

This study had several limitations related to its single center retrospective nature, which is source of selection bias and limits external validity, even though we used a large case-control study design. Firstly, the word recognition query in the 22,671 reports may have slightly underestimated the incidence of artefacts if nuclear medicine physicians did not mention them. Secondly, it resulted in a missing data on the venous catheter caliber used to perfuse the patient, which prevented its inclusion in the analysis of protective and confounding factors for HCa occurrence. As mentioned above, we believe that the paradoxical statistical relationship between proximal (risk factor) and distal (protective factor) venous access could be explained by the use of small-caliber catheters distally to minimize vascular trauma. Thirdly, it also prevented us from studying the effect of injection type (manual versus automatic), as all our patients were injected with an automated system. Further prospective studies are needed to assess the effect of injection type and catheter size on the occurrence of artifacts. Finally, this study was limited to the specific case of FDG, whereas the problem of false-positives results may also concern other radiopharmaceuticals used in PET/CT. For example, Sgard B et al. in 2020 reported a case of pulmonary artifact on PET/CT with prostate-specific membrane antigen (PSMA) radioligands in the setting of biochemical recurrence of prostate adenocarcinoma. They associated this PSMA uptake with vascular malformation, which is different from a hot clot phenomenon [22].

**Table 4:** Review of literature

Reference	Year	Cases (*n*=)	Gender	Age (years)	HCa number	Location	SUVmax	Extravasation
El Yaagoubi et al.	2020	1	F	63	1	NA	26.1	NA
Ozdemir et al.	2014	2	F, M	53, 23	1, 1	RUL, LLL	28.8, 17.7	2 yes
Sánchez-Sánchez et al.	2010	4	2 M, 2 F	77, 37, 64, 70	2, 1, 1, 1	4RUL, 1RLL	185.05, 18.78, 17.51, 31.82, 31.82	3 yes
Fathinul Fikri et al.	2010	1	F	61	1	NA	17.9	NA
Ha et al.	2009	3	2 M, 1 F	66, 71, 75	1, 5, 1	1RUL, 1LM, 3LUL, 2LLL	80, 28.6*, 3.4	NA
Karantanis et al.	2007	3	2 M, 1 F	30, 68, 57	1, 1, 1	NA	NA	NA
Farsad et al.	2005	4	4 F	NA	1, 1, 1, 1	NA	NA	4 yes
Hany et al.	2003	3	3 M	58, 11, 56	1, 1, 1	1RUL, 1LM 1RLL	NA	1 yes

*a single value provided for all 5 HCa NA = not available

## Conclusion

Hot clot artefact is a real but rare phenomenon, occurring in about 1% of examinations and representing a pitfall in the interpretation of 18F-FDG PET scans. The results of our large case-control study suggest that this focal pulmonary tracer uptake is mostly unique, intense and small in volume (< 1 ml); often peripheral in location and associated with the presence of vascular adhesion on images. This supports the hypothesis of a micro embolic origin due to probable trauma to the vessel wall at injection site.

## Data Availability

No datasets were generated or analysed during the current study.
